# Integrating Biological Sex in Precision Psychiatry: The advantage of Using Machine Learning for Personalized Care

**DOI:** 10.1192/j.eurpsy.2025.135

**Published:** 2025-08-26

**Authors:** M. F. Urquijo

**Affiliations:** Dept. for Psychiatry and Psychotherapy, LMU Hospital, Munich, Germany

## Abstract

**Abstract:**

Sex differences in psychiatric disorders are well-documented, yet clinical diagnoses remain primarily symptom-based, overlooking underlying neurobiological distinctions. Despite evidence of sex-specific symptomatology leading to similar diagnostic labels, treatment paradigms often follow a one-size-fits-all approach, contributing to misdiagnosis, suboptimal treatment, and delayed functional recovery. Women, in particular, are disproportionately affected, as psychiatric research has historically prioritized male cohorts to control for hormonal fluctuations and reproductive events (e.g., menarche, pregnancy, menopause), resulting in a gap in sex-specific interventions.

With the advancement of precision psychiatry, integrating sex-informed, multimodal approaches into clinical decision-making is imperative. Machine learning (ML) provides a promising avenue for improving diagnostic accuracy and individualized risk prediction, moving beyond conventional categorical diagnoses.

Here I will highlight findings from two studies leveraging ML to analyze sex-related neuroanatomical patterns:

- Neuroanatomical Sex Differences in Early-Phase Psychiatric Disorders – Investigating grey matter volume alterations in individuals at clinical high risk for psychosis (CHR), recent-onset psychosis (ROP), and recent-onset depression (ROD) using a Support Vector Machine (SVM) model.
- Sex Differences and Neuroanatomical Classification in Transgender Individuals – Exploring whether ML classifiers trained on cisgender populations accurately reflect neurobiological patterns in transgender individuals, considering sex assigned at birth, gender identity, and hormone therapy.

This research does not seek to exclude individuals with Differences in Sex Development (DSD) but rather aims to establish biological sex as a critical, yet underutilized, variable in psychiatric research. Recognizing sex-specific neurobiological mechanisms is a necessary step toward developing targeted risk calculators (e.g., for postpartum depression, suicide risk) and advancing personalized mental health interventions. By refining ML-based models and integrating sex-informed frameworks, this work contributes to the broader goal of precision psychiatry—tailoring psychiatric care to the diverse biological and psychological realities of individuals.

**Disclosure of Interest:**

None Declared

